# Economic evaluation of lymphaticovenous anastomosis *versus* conservative therapy for breast cancer-related lymphoedema: secondary outcome analysis of a randomized clinical trial

**DOI:** 10.1093/bjs/znag062

**Published:** 2026-05-20

**Authors:** Alieske Kleeven, Yasmine M J Jonis, Olivia Currie, Joost Wolfs, Merel Kimman, Hanneke Tielemans, René R W J van der Hulst, Stefan Hummelink, Shan Shan Qiu

**Affiliations:** Department of Plastic, Reconstructive, and Hand Surgery, Maastricht University Medical Centre+, Maastricht, The Netherlands; Department of Plastic Surgery, Radboud University Medical Centre, Nijmegen, The Netherlands; GROW, School for Oncology and Reproduction, Maastricht University, Maastricht, The Netherlands; Department of Plastic, Reconstructive, and Hand Surgery, Maastricht University Medical Centre+, Maastricht, The Netherlands; GROW, School for Oncology and Reproduction, Maastricht University, Maastricht, The Netherlands; Faculty of Health, Medicine, and Life Sciences, Maastricht University, Maastricht, The Netherlands; Department of Plastic, Reconstructive, and Hand Surgery, Maastricht University Medical Centre+, Maastricht, The Netherlands; Department of Clinical Epidemiology and Medical Technology Assessment, Maastricht University Medical Centre+, Maastricht, The Netherlands; Department of Plastic Surgery, Radboud University Medical Centre, Nijmegen, The Netherlands; Department of Plastic, Reconstructive, and Hand Surgery, Maastricht University Medical Centre+, Maastricht, The Netherlands; Department of Plastic Surgery, Radboud University Medical Centre, Nijmegen, The Netherlands; Department of Plastic, Reconstructive, and Hand Surgery, Maastricht University Medical Centre+, Maastricht, The Netherlands

## Abstract

**Background:**

Lymphaticovenous anastomosis (LVA) is an increasingly applied microsurgical option for lymphoedema. The aim of this study was to evaluate the cost-effectiveness of LVA combined with complex decongestive therapy (CDT) *versus* CDT alone for breast cancer-related lymphoedema (BCRL).

**Methods:**

A cost-effectiveness analysis was performed as a pre-specified secondary outcome of an RCT comparing LVA combined with CDT *versus* CDT alone. The primary outcome of the trial was health-related quality of life (HRQoL). A societal perspective with a 2-year time horizon was adopted. Quality-adjusted life-years (QALYs) were derived from the EuroQol five-dimension, five-level (EQ-5D-5L) questionnaire. Uncertainty was assessed using bootstrapping and sensitivity analysis. Data were collected from four Dutch hospitals. The primary outcome was the incremental cost-effectiveness ratio (ICER).

**Results:**

One hundred female patients were included (mean age 58.5 years). Over 2 years, mean total costs were €16 234 (LVA) *versus* €14 293 (CDT); adjusted mean difference €78 (95% c.i. −€6044 to €6200). Mean(s.e.) QALYs were 1.636(0.003) (LVA) *versus* 1.579(0.002) (CDT); adjusted mean difference 0.045 (95% c.i. −0.021 to 0.112). The ICER from a societal perspective was €1716/QALY, with probabilities of cost-effectiveness of 0.60 and 0.71 at the €20 000 and €50 000 willingness-to-pay thresholds respectively. From a healthcare perspective, the ICER was €59 679/QALY, with probabilities of cost-effectiveness of 0.08 and 0.42 at the same thresholds respectively.

**Conclusion:**

LVA combined with CDT was more costly than CDT alone, but societal costs were limited during the 2-year follow-up interval. The LVA group acquired slightly more QALYs. From a societal perspective, LVA combined with CDT has the potential to be cost-effective, particularly when performed under local anaesthesia.

**Registration number:**

NCT02790021 (http://www.clinicaltrials.gov).

## Teaser

First RCT worldwide to evaluate both the clinical effectiveness and cost-effectiveness of lymphaticovenous anastomosis (LVA) combined with complex decongestive therapy (CDT) compared with CDT alone in patients with breast cancer-related lymphoedema (BCRL).

## Introduction

In the Netherlands, one in seven women is diagnosed with breast cancer, accounting for approximately 30% of all cancer diagnoses among women^1^. A common complication after oncological treatment is breast cancer-related lymphoedema (BCRL), with reported incidence rates varying between 5% and 30%, depending on treatment received and individual risk factors^2–4^. BCRL imposes a significant physical and psychological burden on patients^5,6^. It also leads to substantial societal costs, including healthcare costs, informal care, and productivity losses^7,8^.

The current standard of care is complex decongestive therapy (CDT), consisting of compression therapy, manual lymphatic drainage (MLD), skin care, and exercise. However, CDT is labour-intensive, costly, and is a lifelong treatment^9,10^. Lymphaticovenous anastomosis (LVA) is a microsurgical procedure in which lymphatic vessels are connected to nearby veins in the subcutaneous tissue, offering a promising alternative for early-stage lymphoedema of the extremities^11^. Previous studies have shown improvements in health-related quality of life (HRQoL) and limb circumference and volume^11–18^. Despite these potential benefits, high-quality evidence on the cost-effectiveness of LVA is lacking.

To address this gap, the first multicentre RCT was conducted to compare LVA combined with CDT *versus* CDT alone, including a cost-effectiveness analysis to inform reimbursement decisions. The primary outcome of the trial was HRQoL, with interim results at 6 months showing improved HRQoL without significant changes in limb volume^19,20^. At 24 months, clinical outcomes showed greater improvements in HRQoL in the LVA group, with modest reductions in limb volume and more patients reducing or discontinuing compression garment use compared with CDT alone (detailed clinical results will be published separately). Total costs were hypothesized to be lower in the LVA group due to reduced need for CDT, reduced need for primary and informal care, and reduced productivity losses. The results of the cost-effectiveness analysis of the RCT are presented here.

## Methods

This manuscript was written in accordance with the CONSORT guidelines and the Consolidated Health Economic Evaluation Reporting Standards 2022 (CHEERS II) checklist (see the *Supplementary material*)^21,22^. The trial is registered at ClinicalTrials.gov (NCT02790021).

### Trial design

The present economic evaluation was a pre-specified secondary outcome of a multicentre, non-blinded RCT conducted at Maastricht University Medical Centre+ and Radboud University Medical Centre in the Netherlands, the only hospitals performing LVA in the Netherlands at study initiation. Zuyderland Medical Centre and Canisius Medical Centre referred eligible patients. Patients were recruited between January 2019 and January 2023, with 2-year follow-up concluding in January 2025. Blinding was not feasible due to a visible scar after LVA. The study protocol has been published and was approved by the Medical Ethics Review Committee of the University Hospital Maastricht and Maastricht University (azM/UM) on 19 December 2018 (reference number 18.0027647; NL67059.068.18) and approved by the institutional review board of each centre^20^.

### Study population

Trial inclusion criteria were: women aged ≥18 years; diagnosis of primary unilateral breast cancer; treatment for breast cancer with sentinel lymph node biopsy, axillary lymph node dissection, or axillary radiotherapy; early-stage lymphoedema of the arm (stage I–IIa according to the International Society of Lymphology (ISL) classification^23^; see the *Supplementary material*); the presence of viable lymphatic vessels confirmed by indocyanine green (ICG) fluorescence lymphography (see the *Supplementary material*)^24^; and a minimum of 3 months of prior conservative treatment.

Exclusion criteria were: prior lymphatic reconstruction procedures (within the last 10-year interval); recurrent breast cancer; distant metastases; bilateral disease; and congenital lymphoedema.

After written informed consent, patients were randomized (1 : 1) to LVA combined with CDT or CDT alone. The follow-up interval was 2 years.

### Comparators

Patients in the LVA group received one or multiple LVAs in a single procedure at the start of the trial, depending on the number of suitable lymphatic vessels that were found by ICG lymphography. Surgeries were performed under general or local anaesthesia. The local procedures were performed in a minor operating theatre, with patients discharged immediately afterward. No reoperations were performed during the follow-up interval. Patients proceeded with their usual CDT regimen after surgery. The CDT group received their standard CDT regimen without surgical intervention.

### Economic evaluation

The economic evaluation followed the Dutch *Guideline for Economic Evaluations in Healthcare* and was primarily performed from a societal perspective, including treatment, healthcare, out-of-pocket, and productivity costs^25^. A healthcare perspective, limited to treatment and healthcare costs, was also adopted. The time horizon was 2 years, equivalent to trial follow-up. The primary outcome was the incremental cost per quality-adjusted life-year (QALY) gained^26^.

### Outcome measures

#### Cost outcomes

Cost data were converted into 2021 price levels in Euros using the consumer price index (CPI) in the Netherlands^27^. Discount rates of 3% for costs and 1.5% for QALYs were applied after the first year^25^. Mean unit costs are provided in the *Supplementary material*.

#### Intervention costs

In the absence of standardized unit prices, LVA costs were estimated using hospital cost-accounting data. The exact cost breakdown remains confidential. Costs included specialist fees, operating theatre time (surgical time of 150 min), operating room personnel (circulating nurse and scrub nurse), the use of an operating microscope, microsurgical sutures, and a microsurgical instrument set. For LVAs under general anaesthesia, additional costs included anaesthesia-related care (general anaesthesia, anaesthesia nurse, preoperative anaesthesiology screening, and postoperative monitoring in the recovery unit), day-care admission, and perioperative laboratory testing and antibiotics. Preoperative ICG lymphatic mapping and complication management were not included in the intervention costs. Follow-up visits were separately captured in the cost analysis.

#### Healthcare-related costs

CDT resource use was measured by need for MLD sessions and use of compression garments at each follow-up time point to assess whether LVA reduced CDT intensity. CDT treatment costs combined annual fitting costs of a new compression garment with self-reported costs and MLD sessions, using standardized unit prices. Data were collected using a modified Institute for Medical Technology Assessment (iMTA) Medical Consumption Questionnaire (iMCQ) with a 3-month recall interval, sent online at 3, 6, 9, 12, 15, 18, and 24 months^28^.

Intermittent pneumatic compression (IPC) is a home-based treatment method that uses an inflatable cuff to apply rhythmic pressure to the arm, thereby stimulating lymphatic drainage and reducing swelling^29,30^. IPC device costs were calculated from the device price obtained from the supplier, expected economic lifespan (5 years), and estimated average usage (2–4 weeks/month). In the Netherlands, IPC devices are typically borrowed, but each patient received a personal arm cuff for hygienic reasons, representing a one-time cost per user. Costs were calculated per hour of use based on annual depreciation over the device lifespan^31^.

Other healthcare costs, including visits to the emergency department, medical specialists, general practitioners (GPs), and other health professionals, as well as hours of home care, were estimated using standardized Dutch unit prices^31^.

#### Costs for patients and family (informal care, out of pocket)

Patient and family costs included hours of informal care (valued using standardized unit prices) and out-of-pocket costs for travel, parking, personal care products, clothing (as regular clothing sometimes does not fit due to extensive swelling of the arm), pain medication, gym memberships, and other lymphoedema-related costs. These costs were entered as direct monetary amounts by patients in the online patient survey.

#### Lost productivity costs

Lost productivity costs were assessed using an adapted iMTA Productivity Cost Questionnaire (iPCQ)^28^ as part of an online survey that was sent out at baseline and at 3, 6, 9, 12, 15, 18, and 24 months. This captured absenteeism, with a distinction made between short-term absence (≤4 weeks) and long-term absence (>4 weeks). For long-term absenteeism, the friction cost approach was applied, assuming that productivity losses are limited to the time required to replace the absent worker that is set at 85 days in the Netherlands^25,32^. Additionally, productivity losses due to presenteeism were included, referring to the hours in which the patient was at work but performed at reduced productivity due to illness. Standardized unit costs were applied to hours of productivity loss^31^.

#### QALYs

HRQoL was measured using the EuroQol five-dimension, five-level (EQ-5D-5L) questionnaire at baseline and at 3, 6, 12, 18, and 24 months. The EQ-5D-5L questionnaire covers problems in mobility, self-care, usual activities, pain/discomfort, and anxiety/depression^33–35^. Responses were converted into utility scores using the Dutch tariff ranging from −0.45 (for health worse than death) to 1 (full health)^36^. QALYs were then calculated by multiplying time spent in each health state by these utility scores.

### Statistical analysis

Statistical analysis was performed in RStudio 2021 (R Foundation for Statistical Computing, Vienna, Austria) between March 2025 and May 2025. For baseline characteristics, continuous variables are summarized as mean(s.d.) and categorical variables are summarized as *n* (%).

The cost-effectiveness analysis followed the intention-to-treat principle. Total costs and QALYs were calculated per patient. Missing data at item level (for example, a patient reported to have received home care or visited a physiotherapist but did not report the number of hours or visits) were resolved by making assumptions based on means per group and other time points. For missing questionnaires, utility and cost data were imputed using multiple imputation by chained equations with predictive mean matching, accounting for skewed distribution of costs^37,38^. The data set was split by treatment group (that is LVA *versus* CDT) before imputation, as recommended by Faria *et al*.^39^. The imputation model included patient characteristics (for example age), clinical characteristics (for example ICG stage), and the outcome variables at other time points. Fifteen data sets were imputed, based on the percentage of missing outcome variables, and analysed separately; results were pooled using Rubin’s Rules^40^.

Mean cost and effect differences between groups were estimated using linear regression models, adjusted for baseline differences in costs and utilities, age, and ICG stage^41^. The incremental cost-effectiveness ratio (ICER) was calculated by dividing mean cost differences by mean effect differences. Uncertainty was assessed through non-parametric bootstrapping with 5000 replications and results were plotted on a cost-effectiveness plane. Finally, cost-effectiveness acceptability curves (CEACs) were generated to show the probability of cost-effectiveness across various willingness-to-pay (WTP) thresholds. In the Netherlands, the WTP threshold typically ranges from €20 000 to €80 000, depending on the disease burden. For this population, the Dutch WTP threshold was set at €20 000^42^.

### Sensitivity analyses

Three sensitivity analyses tested the robustness of the results. First, the analysis was repeated without baseline correction, as recommended by the Dutch guidelines^25^. Second, LVA costs were recalculated assuming all procedures used local anaesthesia (€3127 per procedure), rather than the combination of local and general anaesthesia used in the base case (€3748 per procedure), reflecting future hospital practice^43^. Third, a scenario was performed assuming all procedures were performed under general anaesthesia (€4369 per procedure).

### Patient involvement

The Dutch Network for Lymphoedema and Lipoedema (NL Net)^44^, along with the Patient Advocacy Group that is a joint initiative from the Breast Cancer Research Group of the Dutch Breast Cancer Association^45^ reviewed and provided feedback on the trial protocol before the start of the trial.

## Results

### Participants

**Figure 1:**
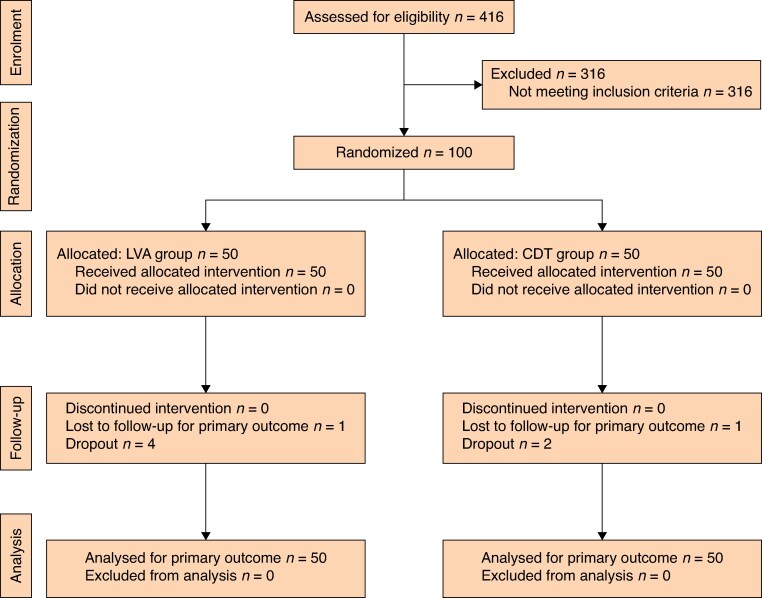
CONSORT flow diagram A total of 100 patients were randomly assigned to either the LVA group or the CDT group in a 1 : 1 ratio. LVA, lymphaticovenous anastomosis; CDT, complex decongestive therapy.

A total of 100 patients were randomized into one of the two groups, with a mean(s.d.) age of 58.5(8.8) years. Patient characteristics are shown in *Table 1*. Two patients were lost to follow-up, and six patients were considered dropouts (including three deaths and three patients withdrew due to the need for more urgent surgical intervention, personal reasons, or dissatisfaction with allocation (*Fig. 1*). The differences in patient numbers compared with earlier time points are attributable to some patients dropping out or being lost to follow-up after the initial 12-month assessment. While baseline data were complete, missing follow-up data at the 24-month mark were addressed through imputation.

**Table 1:** Baseline characteristics

	LVA group	CDT group
Number of patients	50 (50)	50 (50)
Age (years), mean(s.d.)	57.7(8.4)	59.4(9.3)
BMI (kg/m^2^), mean(s.d.)	26(3.47)	28(5.21)
Lymphoedema onset (months), mean(s.d.)	81(73)	82(68)
**Affected arm**		
Left	29 (58)	30 (60)
Right	21 (42)	20 (40)
Current smoker	2 (4)	1 (2)
**Previous breast cancer treatment**		
Radiotherapy of the breast	45 (90)	45 (90)
Radiotherapy of the axilla	43 (86)	44 (88)
Sentinel node procedure	26 (52)	24 (48)
Axillary lymph node dissection	49 (98)	49 (98)
Chemotherapy	46 (92)	48 (96)
Hormone therapy	35 (70)	33 (66)
Complications	15 (30)	13 (26)
**ISL stage**		
I	1 (2)	2 (4)
IIa	49 (98)	48 (96)
**ICG stage**		
II	6 (12)	11 (22)
III	44 (88)	39 (78)
**Conservative therapy**		
Use of compression garment	47 (94)	42 (84)
MLD	43 (86)	40 (80)
Use of IPC	7 (14)	5 (10)
**Highest education**		
Primary school	0 (0)	1 (2)
High school	11 (22)	14 (28)
Vocational education	11 (22)	15 (30)
Higher professional education	20 (40)	16 (32)
University	8 (16)	4 (8)
**Daily occupation**		
Employed	30 (60)	26 (52)
Retired	10 (20)	11 (22)
Unemployed	2 (4)	7 (14)
Partly unemployed due to health issues	4 (8)	5 (10)
Other	4 (8)	1 (2)
Sick leave due to lymphoedema*†	5 (10)	3 (6)
Longer than the past 4 weeks	1 (2)	2 (4)
Reduced productivity due to health issues*‡	18 (36)	13 (26)
EQ-5D-5L utility value, mean(s.e.)	0.783(0)	0.753(0.0004)

Values are *n* (%) unless otherwise indicated. *In the past 4 weeks before the questionnaire. †Absenteeism. ‡Presenteeism. LVA, lymphaticovenous anastomosis; CDT, complex decongestive therapy; ISL, International Society of Lymphology; ICG, indocyanine green; MLD, manual lymphatic drainage; IPC, intermittent pneumatic therapy; EQ-5D-5L, EuroQol five-dimension, five-level.

### Costs

The response rate for the combined iMCQ and iPCQ was 88.9%. Over 2 years, mean societal costs were €16 234 for LVA combined with CDT *versus* €14 293 for CDT alone, with an adjusted mean difference of €78 (bootstrapped 95% c.i. −€6044 to €6200). The cost analysis per treatment group is shown in *Table 2*. CDT showed higher costs in primary and informal care.

**Table 2:** Effects and costs per treatment group

	Mean(s.e.)		Adjusted mean difference (95% c.i.)
	LVA group	CDT group	
**EQ-5D-5L index scores**			
Baseline	0.783(0)	0.753(0.0004)	
3 months	0.814(0.001)	0.768(0.001)	
6 months	0.825(0.001)	0.765(0.001)	
12 months	0.830(0.003)	0.786(0.002)	
18 months*	0.826(0.002)	0.815(0.003)	
24 months*	0.789(0.002)	0.820(0.001)	
EQ-5D-5L QALYs	1.636(0.003)	1.579(0.002)	0.045 (−0.021,0.112)
**Healthcare-related costs**			
LVA surgery costs	3748	0	
Compression therapy	1449(14)	1157(5)	
Hospital costs	901(12)	596(8)	
Primary care (GP, home care, allied HCPs)	935(8)	2084(34)	
MLD care	1124(3)	1221(7)	
Total healthcare costs	8157(20)	5059(41)	2647 (1028,4266)
**Costs for patients and family**			
Informal care costs	1001(14)	3090(30)	
Out-of-pocket costs	725(4)	789(4)	
**Lost productivity costs**			
Absenteeism and presenteeism	4494(56)	3578(54)	
Unpaid work	1857(20)	1777(24)	
Total societal costs	16 234(65)	14 293(59)	78 (−6044,6200)

^*^Discounted values. LVA, lymphaticovenous anastomosis; CDT, complex decongestive therapy; EQ-5D-5L, EuroQol five-dimension, five-level; QALYs, quality-adjusted life years; GP, general practitioner; HCPs, allied healthcare professionals; MLD, manual lymphatic drainage.

CDT-related resource use showed a slight decrease in MLD costs over time in the LVA group from €197 at baseline to €111 at 24 months compared with the CDT group from €168 at baseline to €120 at 24 months. Differences were modest and not statistically significant, suggesting that LVA did not substantially reduce CDT utilization over the 2-year follow-up interval.

### Effects

The total response rate of the EQ-5D-5L questionnaire was 89.9%. After 2 years, the (adjusted) QALY difference was 0.045 in favour of the LVA group (95% c.i. −0.021 to 0.112).

### Cost-effectiveness

**Figure 2:**
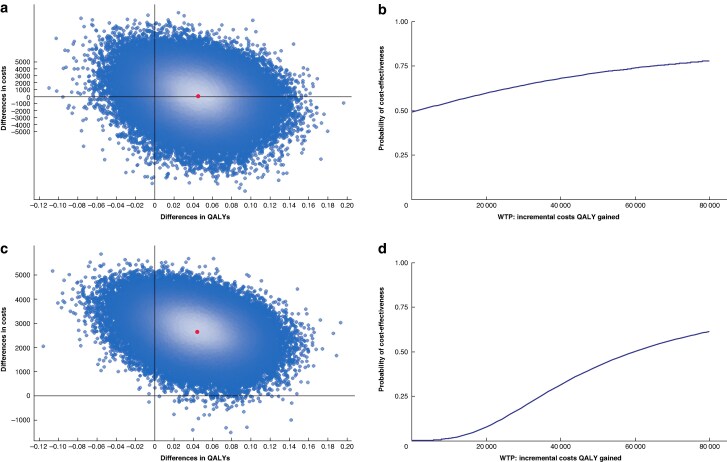
Cost-effectiveness planes and CEACs for LVA combined with CDT *versus* CDT alone over a 2-year time horizon from societal and healthcare perspectives **a** and **c** Cost-effectiveness planes presenting a comparison between LVA combined with CDT and CDT alone, from both a societal perspective (**a**) and a healthcare perspective (**c**). **b** and **d** CEACs presenting a comparison between LVA combined with CDT and CDT alone, from both a societal perspective (**b**) and a healthcare perspective (**d**). Cost-effectiveness planes and CEACs were used to evaluate QALYs. The cost-effectiveness planes illustrate point estimates of the ICER (central dot) surrounded by bootstrap replications. The CEACs display the probability that LVA combined with CDT is cost-effective compared with CDT alone across a range of WTP thresholds per QALY gained; WTP thresholds are given in Euros. CEACs, cost-effectiveness acceptability curves; LVA, lymphaticovenous anastomosis; CDT, complex decongestive therapy; QALYs, quality-adjusted life-years; ICER, incremental cost-effectiveness ratio; WTP, willingness-to-pay.

Dividing the adjusted mean cost difference (€78) by the QALY difference (0.045) resulted in an ICER of €1716 (*Table 3*). On the cost-effectiveness plane of the societal perspective (*Fig. 2a*), most bootstrap replications lay in the north-east quadrant (46%). This suggests that LVA combined with CDT is more effective and slightly more expensive than CDT alone. Another 44% appeared in the south-east quadrant, indicating that LVA combined with CDT is more effective but less costly (dominant), although the cost estimates have relatively wide 95% confidence intervals. The CEAC showed a 0.60 probability of cost-effectiveness at €20 000 per QALY and a 0.71 probability of cost-effectiveness at €50 000 per QALY (*Fig. 2b*).

**Table 3:** Results of the cost-effectiveness and cost-utility analysis

ΔC (95% c.i.)	ΔE (95% c.i.)	ICER	Cost-effectiveness plane				Probability of cost-effectiveness	
			NE (%)	SE (%)	SW (%)	NW (%)	at €20 000	at €50 000
**Societal perspective (main analysis)**								
78 (−6044,6200)	0.045 (−0.021,0.112)	1716	46	44	3	7	0.60	0.71
**Healthcare perspective**								
2647 (1028,4266)	0.044 (−0.022,0.111)	59 679	90	0	0	10	0.08	0.42
**Sensitivity analysis 1: no corrections for baseline differences**								
1941 (−4647,8530)	0.057 (−0.025,0.140)	33 779	65	27	0	8	0.42	0.58
**Sensitivity analysis 2: LVA only under local anaesthesia**								
−387 (−5724,4949)	0.045 (−0.021,0.112)	Dominant	40	51	3	6	0.67	0.77
**Sensitivity analysis 3: LVA only under general anaesthesia**								
680 (−5464,6842)	0.045 (−0.021,0.112)	14 997	53	37	2	7	0.53	0.66

ΔC, difference in costs; ΔE, difference in effectiveness; ICER, incremental cost-effectiveness ratio; NE, north-east; SE, south-east; SW, south-west; NW, north-west; LVA, lymphaticovenous anastomosis.

From a healthcare perspective, the ICER was €59 679, with probabilities of cost-effectiveness of 0.08 and 0.42 at the same thresholds respectively (*Fig. 2d*).

### Sensitivity analysis

Three sensitivity analyses were performed (*Table 3*). The sensitivity analysis without baseline corrections showed higher costs compared with the main analysis, leading to a lower probability of cost-effectiveness. LVA combined with CDT remained likely to be more effective than CDT alone, but also more expensive.

The sensitivity analysis assuming all LVA procedures were performed solely under local anaesthesia showed increased probabilities of cost-effectiveness, with most bootstrap replications in the south-east quadrant (more effective, less costly). However, in the present trial, 33 of 50 LVAs were performed under local anaesthesia and 17 were performed under general anaesthesia, according to the different protocols used at the hospitals.

The sensitivity analysis assuming all LVA procedures were performed under general anaesthesia resulted in higher intervention costs compared with the base-case analysis, yielding an ICER of €14 997 per QALY gained. Most bootstrap replications were in the north-east quadrant (more effective, more costly). The probability of cost-effectiveness decreased slightly compared with the main analysis.

## Discussion

This study represents the first RCT reporting on cost-effectiveness of LVA combined with CDT *versus* CDT alone over 2 years. From a societal perspective, LVA combined with CDT appeared more effective but slightly more costly, with an ICER of €1716 per QALY gained and a 0.60 probability of cost-effectiveness at the Dutch WTP threshold of €20 000 per QALY. This combined approach may be considered a potentially cost-effective intervention, though with considerable uncertainty. Only modest reductions in MLD were observed in the LVA group compared with CDT alone, while compression garment and bandage use remained similar, indicating cost-effectiveness was primarily driven by QALY gains.

From a healthcare perspective, the ICER was substantially higher (€59 679) with a low probability of cost-effectiveness, reflecting high upfront surgical costs without immediate offsetting savings, highlighting that reimbursement decisions based solely on the healthcare budget perspective may not support widespread adoption. Sensitivity analyses showed that results were sensitive to baseline correction and surgical costs; when all procedures were assumed to be performed exclusively under local anaesthesia, LVA became dominant.

The findings are consistent with prior smaller-scale or model-based studies suggesting LVA may be a cost-effective or even cost-saving treatment modality. A prospective single-centre study published in 2025, involving 150 patients, reported reduced costs related to cellulitis (such as hospitalizations) in patients with lymphoedema^46^; the study also found improved quality of life (EQ-5D-5L questionnaire) and an ICER of £54 231 after 2 years, indicating that LVA was more effective but also more costly, which corroborates the present findings. A recent cost-minimization analysis found that prophylactic LVA after mastectomy with axillary lymph node dissection resulted in lifetime savings of $7646.65 (45.2%) per patient^47^. Although not directly comparable, these findings suggest LVA may have favourable economic outcomes in different clinical contexts. A model-based cost analysis further reported that the overall costs for LVA and CDT were comparable, as the higher surgical expenses for LVA were offset by savings from reduced ongoing CDT^48^. The observed improvement in HRQoL aligns with previous studies reporting improved patient-reported outcomes and functional status after LVA, underlining its clinical relevance beyond cost considerations^12,14,49–51^.

Given the favourable societal cost-effectiveness, policymakers and insurers may consider reimbursement of LVA, especially when performed under local anaesthesia in patients meeting the inclusion criteria of the present trial. LVA material costs are largely fixed, but shorter operating times could reduce personnel and theatre expenses. Efficiency depends on surgeon experience, suggesting that LVA may be most efficiently performed in specialized and centralized centres.

Strengths of the trial include its multicentre design, 2-year follow-up, patient-level societal cost data, and adherence to the Dutch health economic guidelines and recommended statistical methods, including multiple imputation and adjustment for baseline differences^25^.

Limitations include the variability in cost data, often skewed and influenced by outliers, resulting in wide 95% confidence intervals for the incremental cost differences, despite multiple imputation for missing data. The 2-year follow-up limits conclusions on long-term costs and effects of LVA combined with CDT *versus* CDT alone, and no formal modelling was performed to extrapolate beyond the trial interval. Furthermore, generalizability may be restricted by Dutch-specific methodological choices (friction cost approach, unit costs, and EQ-5D-5L tariff), varying WTP thresholds, and hospital-specific LVA costs; requiring setting-specific inputs to assess cost-effectiveness in other contexts. Finally, although CDT adherence was recorded, no formal subgroup analyses assessed whether differential adherence or socioeconomic factors influenced costs or outcomes, as such analyses generally do not inform reimbursement decision-making.

This multicentre RCT demonstrates that LVA combined with CDT was more effective but slightly more costly than CDT alone for the treatment of BCRL over 2 years. Modest reductions in MLD were observed in the LVA group, while compression garment and bandage use remained similar, indicating that cost-effectiveness was mainly driven by QALY gains. From a healthcare perspective, LVA combined with CDT is not cost-effective at common thresholds. From a societal perspective, LVA has the potential to be cost-effective, particularly when performed under local anaesthesia, although considerable uncertainty remains.

## Supplementary Material

znag062_Supplementary_Data

## Data Availability

Data will not be made available.
