# Supplementary material for: Economic evaluation of lymphaticovenous anastomosis versus conservative therapy for breast cancer-related lymphoedema: secondary outcome analysis of a randomized clinical trial
Source: Br J Surg. 2026 May 20;113(6):znag062. doi: 10.1093/bjs/znag062 (PMC13289813; doi:10.1093/bjs/znag062)
Supplement: znag062_Supplementary_Data [file znag062_supplementary_data.docx]

**Economic evaluation of lymphaticovenous anastomosis versus conservative therapy for breast-cancer related lymphoedema: secondary outcome analysis of a randomized controlled trial**

Alieske Kleeven, MD^1,2,3^; Yasmine M.J. Jonis^1,3^, MD; Olivia Currie^4^; Joost Wolfs, MD^1^, PhD; Merel Kimman, PhD^5^; Hanneke Tielemans, MD^2^; René R.W.J. van der Hulst, MD, PhD^1^; Stefan Hummelink, PhD^2^; Shan Shan Qiu, MD, PhD^1^

**Affiliations**

^1^ Department of Plastic, Reconstructive, and Hand Surgery, Maastricht University Medical Center+, Maastricht, the Netherlands.

^2^ Department of Plastic Surgery, Radboud University Medical Center, Nijmegen, the Netherlands.

^3^ GROW, School for Oncology and Reproduction, Maastricht University, the Netherlands.

^4^ Faculty of Health, Medicine, and Life Sciences, Maastricht University, Maastricht, the Netherlands.

^5^ Department of Clinical Epidemiology and Medical Technology Assessment, Maastricht University Medical Center+, Maastricht, the Netherlands.

**Corresponding author:**

Shan Shan Qiu, MD, PhD

Department of Plastic, Reconstructive, and Hand Surgery

Maastricht University Medical Center+

P. Debyelaan 25, 6229 HX, Maastricht, the Netherlands

[Shanshan.qiushao@mumc.nl](mailto:Shanshan.qiushao@mumc.nl)

+31(0)43-3877481

**Supplementary Materials - Index**

| **Supplementary Methods** |  |
| --- | --- |
| S2. International Society of Lymphology (ISL) classification for lymphedema | *pag. 2* |
| S3. Indocyanine green (ICG) classification for arm lymphedema | *pag. 2* |
| **Supplementary Results** |  |
| N/A |  |
| **Supplementary Appendixes** |  |
| S1. CHEERS 2022 Checklist | *pag. 4* |
| **Supplementary Figures and Tables** |  |
| S4. Mean costs per unit | *pag. 5* |
| **References** | *pag. 7* |
|  |  |

Supplementary material is labeled based on its first mention in the article.

**Supplementary Methods**

**S1. International Society of Lymphology (ISL) classification for lymphedema**

The ISL classification for lymphedema is a widely used clinical staging system that describes the severity and progression of lymphedema. Stages are based on clinical presentation, reversibility, and tissue changes.

- **Stage 0:** A latent or subclinical condition where swelling is not yet evident despite impaired lymph transport, subtle alterations in tissue fluid/composition, and changes in subjective symptoms. It can be transitory and may exist months or years before overt edema occurs.
- **Stage I**: Represents an early accumulation of fluid relatively high in protein content which subsides with limb elevation. Pitting may occur. An increase in various types of proliferating cells may also be seen.
- **Stage IIa**: More changes in solid structures, limb elevation alone rarely reduces tissue swelling, and pitting is manifest.
- Stage IIb: The limb may not pit as excess subcutaneous fat and fibrosis develop.
- Stage III: Lymphostatic elephantiasis where pitting can be absent and thropic skin character and thickness, further deposition of fat and fibrosis, and warty overgrowths have developed^1^.

**S2. Indocyanine green (ICG) classification for arm lymphedema**

The ICG classification is a system used to stage lymphedema based on indocyanine green (ICG) fluorescence lymphography patterns. It assesses the severity of lymphatic dysfunction by visualizing lymphatic flow and dermal backflow patterns. The classification includes five stage (0 to IV):

• Stage 0: No edema or dermal backflow pattern is seen on ICG green lymphography.

• Stage 1: A splash pattern is observed around the axilla.

• Stage II: The startdust pattern is limited proximally to the olecranon.

• Stage III: The startdust pattern exceeds the olecranon.

• Stage IV: The startdust pattern is observed throughout the limb.

• Stage V: A diffuse pattern becomes evident with presence of the startdust pattern^2^.

**Supplementary Results**

N/A.

**Supplementary Appendixes**

S1. CHEERS II Checklist

|  | **Item** | **Guidance for Reporting** | **Reported in section** |
| --- | --- | --- | --- |
| **TITLE** | | |  |
| Title | 1 | Identify the study as an economic evaluation and specify the interventions being compared. | Title (p. 1) |
| **ABSTRACT** | | |  |
| Abstract | 2 | Provide a structured summary that highlights context, key methods, results and alternative analyses. | ­­Abstract (p. 2) |
| **INTRODUCTION** | | |  |
| Background and objectives | 3 | Give the context for the study, the study question and its practical relevance for decision making in policy or practice. | Introduction (p. 4) |
| **METHODS** | | |  |
| Health economic  analysis plan | 4 | Indicate whether a health economic analysis plan was developed and  where available. | Methods – trial design (p. 5) |
| Study population | 5 | Describe characteristics of the study population (such as age range, demographics, socioeconomic, or clinical characteristics). | Methods – study population (p. 5,6) |
| Setting and location | 6 | Provide relevant contextual information that may influence findings. | Methods (p. 5-10) |
| Comparators | 7 | Describe the interventions or strategies being compared and why chosen. | Methods – comparators (p.6) |
| Perspective | 8 | State the perspective(s) adopted by the study and why chosen. | Methods – economic evaluation (p.6) |
| Time horizon | 9 | State the time horizon for the study and why appropriate. | Methods – economic evaluation (p.6) |
| Discount rate | 10 | Report the discount rate(s) and reason chosen. | Methods – economic evaluation (p. 6) |
| Selection of outcomes | 11 | Describe what outcomes were used as the measure(s) of benefit(s) and harm(s). | Methods – outcome measures (p.6-9) |
| Measurement of outcomes | 12 | Describe how outcomes used to capture benefit(s) and harm(s) were measured. | Methods – outcome measures (p.6-9) |
| Valuation of outcomes | 13 | Describe the population and methods used to measure and value outcomes. | Methods – outcome measures (p.6-9) |
| Measurement and valuation of resources  and costs | 14 | Describe how costs were valued. | Methods – cost outcomes (p.6-8) |
| Currency, price date, and conversion | 15 | Report the dates of the estimated resource quantities and unit costs, plus the currency and year of conversion. | Methods – cost outcomes (p.6-8) |
| Rationale and  description of model | 16 | If modelling is used, describe in detail and why used. Report if the model  is publicly available and where it can be accessed. | N/A |
| Analytics and assumptions | 17 | Describe any methods for analysing or statistically transforming data, any extrapolation methods, and approaches for validating any model used. | N/A |
| Characterizing heterogeneity | 18 | Describe any methods used for estimating how the results of the study vary for sub-groups. | N/A |
| Characterizing  distributional effects | 19 | Describe how impacts are distributed across different individuals  or adjustments made to reflect priority populations. | N/A |
| Characterizing uncertainty | 20 | Describe methods to characterize any sources of uncertainty in the analysis. | Methods – statistical analysis (p.9,10) |
| Approach to engagement with patients and others affected by the study | 21 | Describe any approaches to engage patients or service recipients, the general public, communities, or stakeholders (e.g., clinicians or payers) in the design of the study. | Methods – patient involvement (p.10) |
| **RESULTS** | | |  |
| Study parameters | 22 | Report all analytic inputs (e.g., values, ranges, references) including uncertainty or distributional assumptions. | Results (p.10,12) |
| Summary of main results | 23 | Report the mean values for the main categories of costs and outcomes of interest and summarise them in the most appropriate overall measure. | Results – table 2 |
| Effect of uncertainty | 24 | Describe how uncertainty about analytic judgments, inputs, or projections  affect findings. Report the effect of choice of discount rate and time horizon, if applicable. | Results – sensitivity analysis (p.12) |
| Effect of engagement with patients and others affected by the study | 25 | Report on any difference patient/service recipient, general public, community, or stakeholder involvement made to the approach or findings of the study | N/A |
| **DISCUSSION** | | |  |
| Study findings, limitations, generalizability, and current knowledge | 26 | Report key findings, limitations, ethical or equity considerations not captured, and how these could impact patients, policy, or practice. | Discussion (p.12,14) |
| **OTHER RELEVANT INFORMATION** | | | |
| Source of funding | 27 | Describe how the study was funded and any role of the funder in the identification, design, conduct, and reporting of the analysis | Source of funding (p.15) |
| Conflicts of interest | 28 | Report authors conflicts of interest according to journal or  International Committee of Medical Journal Editors requirements. | Conflicts of interest (p.14)­­ |

**Supplementary Figures and Tables**

S3. Mean costs per unit

| **Cost category** | | **Unit cost (€)** | **Description** | **Source** |
| --- | --- | --- | --- | --- |
| LVA surgery costs | |  |  |  |
|  | *LVA (average)* | 3748 | Per procedure | Hospital microcosting^a^ |
|  | *LVA (under general anesthesia)* | 4369 | Per procedure | Hospital microcosting^a^ |
|  | *LVA (under local anesthesia)* | 3127 | Per procedure | Hospital microcosting^a^ |
| CDT | |  |  |  |
|  | *Arm compression stocking fitting* | 136 | Per fitting | Hospital microcosting^a^ |
|  | *Arm compression stocking* | 45 | Per item | Hospital microcosting^a^ |
|  | *Arm compression bandages* | 27 | Per set | Market estimate^b^ |
|  | *Manual lymphatic drainage visit* | 55 | Per session | Hospital microcosting^a^ |
| IPC | |  |  |  |
|  | *IPC pump* | 2.356 | Per item | Market estimate^b^ |
|  | *IPC arm sleeve* | 536 | Per item | Market estimate^b^ |
|  | *IPC usage* | 16 | Per hour | Estimate ^c^ |
| Other healthcare-related costs | |  |  |  |
|  | *Outpatient clinic* | 109 | Per visit | Dutch costing guidelines (2024)^c^ |
|  | *Emergency department* | 235 | Per visit | Dutch costing guidelines (2024)^c^ |
|  | *General practitioner* | 28 | Per visit | Dutch costing guidelines (2024)^c^ |
|  | *Social worker* | 115 | Per session | Dutch costing guidelines (2024)^c^ |
|  | *Mental health therapist* | 110 | Per session | Dutch costing guidelines (2024)^c^ |
|  | *Other therapist (e.g. physical therapist not part of CDT)* | 35 | Per session | Dutch costing guidelines (2024)^c^ |
|  | *Occupational therapist* | 22 | Per session | Dutch costing guidelines (2024)^c^ |
|  | *Dietitian* | 22 | Per consultation | Dutch costing guidelines (2024)^c^ |
|  | *Occupational physician* | 109 | Per consultation | Dutch costing guidelines (2024)^c^ |
|  | *Alternative medicine* | 22 | Per session | Dutch costing guidelines (2024)^c^ |
|  | *Home care* | 52 | Per hour | Dutch costing guidelines (2024)^c^ |
| Patient and family costs | |  |  |  |
|  | *Informal care* | 17 | Per hour | Dutch costing guidelines (2024)^c^ |
|  | *Unpaid work loss* | 17 | Per hour | Dutch costing guidelines (2024)^c^ |
| Indirect costs (productivity loss) | |  |  |  |
|  | *Productivity loss* | 36 | Per hour | Dutch costing guidelines (2024)^c^ |

Abbreviations: LVA, lymphaticovenous anastomosis; CDT, complex decongestive therapy; IPC, intermittent pneumatic compression.

^a^ Based on detailed microcosting at Maastricht University Medical center+ (MUMC+) and Radboud University Medical center (Radboudumc).

^b^ Based on prices from suppliers.

^c^ Reference prices from the Dutch costing manual from the Dutch health economic guidelines^3,4^.

**References**

1. Executive C. The Diagnosis and Treatment of Peripheral Lymphedema: 2016 Consensus Document of the International Society of Lymphology. *Lymphology*. Dec 2016;49(4):170-84.
2. Yamamoto T, Yamamoto N, Doi K, Oshima A, Yoshimatsu H, Todokoro T, et al. Indocyanine green-enhanced lymphography for upper extremity lymphedema: a novel severity staging system using dermal backflow patterns. Plast Reconstr Surg. 2011;128(4):941-7.
3. Institute DNHC. *Guideline for economic evaluations in healthcare.* 2024 <https://english.zorginstituutnederland.nl/publications/reports/2024/01/16/guideline-for-economic-evaluations-in-healthcare>
4. Institute DNHC. *Costing manual* 2024 <https://english.zorginstituutnederland.nl/publications/reports/2024/01/16/guideline-for-economic-evaluations-in-healthcare>
